# Navigating Barriers to Dental Care for Patients with Down Syndrome: A Scoping Review of Challenges and Strategies

**DOI:** 10.3390/children12030330

**Published:** 2025-03-05

**Authors:** Man Hung, Aaron Graves, Johanna Lu, Connor Schwartz, Martin S. Lipsky

**Affiliations:** 1 Department of Orthopaedic Surgery Operations, University of Utah, Salt Lake City, UT 84108, USA; 2 Division of Public Health, University of Utah, Salt Lake City, UT 84108, USA; 3 College of Dental Medicine, Roseman University of Health Sciences, South Jordan, UT 84095, USA; 4 The Wharton School, University of Pennsylvania, Philadelphia, PA 19104, USA; 5 Veteran Affairs Salt Lake City Health Care, Salt Lake City, UT 84148, USA; 6 Huntsman Cancer Institute, Salt Lake City, UT 84112, USA; 7 Department of Chemistry and Biochemistry, Weber State University, Ogden, UT 84408, USA; 8 Library, Roseman University of Health Sciences, South Jordan, UT 84095, USA; 9 Institute on Aging, Portland State University, Portland, OR 97201, USA

**Keywords:** Down syndrome, dental care, barriers, special needs dentistry, preventive strategies

## Abstract

Objective: Access to dental care is critical for overall health, yet individuals with Down Syndrome (DS) face numerous barriers, including communication difficulties, insufficiently trained professionals, and financial constraints leading to poor oral health outcomes. These obstacles are compounded by a shortage of specialized services and geographic disparities that limit access to appropriate care. This scoping review aimed to explore the existing literature about these barriers and to identify strategies to enhance dental care for this vulnerable population. Methods: This scoping review followed the Systematic Reviews and Meta-Analyses extension for Scoping Review guidelines. A comprehensive search was conducted across PubMed, Scopus, and Web of Science focusing on peer-reviewed studies published in English within the last 10 years that examined barriers and strategies related to patients with DS. To ensure methodological rigor, eligible studies were independently screened and reviewed by two reviewers. Any disagreements were resolved through discussion, and if consensus could not be reached, a third reviewer made the final decision. Data were extracted using a standardized form. The extracted data were analyzed and synthesized to identify and categorize barriers and strategies across different studies. Results: The initial search yielded 58 articles, with 42 remaining after duplicates were removed. Following titles and abstracts screening, 13 studies were considered for full-text review, and 6 studies met the inclusion criteria. These studies, published between 2015 and 2023, primarily employed cross-sectional designs and identified key barriers, including challenges in maintaining oral hygiene, limited access to specialized dental services, and behavioral and sensory difficulties affecting dental care for children with DS. Strategies to overcome these barriers included enhancing dental professional training, developing tailored care approaches, and integrating preventive dental programs into broader health initiatives. Conclusions: This review highlights significant, persistent barriers to dental care for children with DS. By emphasizing the need for enhanced dental professional training, personalized care approaches, and integrated preventive programs, this review provides a framework for improving dental care accessibility for this population. Addressing these barriers can lead to better oral health outcomes and improved overall well-being for children with DS.

## 1. Introduction

Access to dental care is a critical aspect of overall health [1,2], yet it remains a major challenge for many vulnerable populations, particularly individuals with Down Syndrome (DS). Patients with DS often encounter barriers that impede their access to timely and high-quality dental care [3]. These barriers are multifaceted [4,5], encompassing patient-related factors, healthcare system limitations, and societal obstacles that collectively contribute to disparities in oral health outcomes.

DS is the most prevalent chromosomal condition in the US, occurring in approximately 1 in 750 live births [6]. Individuals with DS are at a disproportionately higher risk of poor oral health compared to the general population [7,8] with higher rates of untreated caries, periodontal disease, and other oral health conditions that can affect their health and quality of life. Additionally, the burden of oral disease in these patients is compounded by various challenges, including difficulties in communication, behavioral management issues, and the need for sedation or general anesthesia during routine dental procedures [9].

A major barrier to dental care for patients with DS is the lack of specialized expertise among dental professionals [10]. Many dentists report feeling unprepared to treat patients with DS due to limited exposure and inadequate training during their education [3]. This lack of confidence and clinical competence can lead to reluctance to accept patients with DS, further exacerbating the issue of access. Moreover, many dental clinics are not adequately equipped to accommodate the unique needs of patients with DS, creating additional obstacles [11].

Financial constraints also play a critical role in limiting access to dental care for patients with DS [9]. Many individuals with DS depend on public health programs, which may not adequately cover necessary dental services or face challenges navigating complex insurance systems to obtain coverage [12]. Furthermore, geographic disparities in access to specialized dental care may mean that patients in rural or underserved areas must travel long distances to receive care [13], adding to the financial and logistical burden.

The COVID-19 pandemic further exacerbated these barriers by restricting access to routine dental services, delaying treatments, and increasing anxiety for children with DS due to disrupted healthcare routines. Many dental clinics faced temporary closures, workforce shortages, and enhanced infection control protocols, making it even more challenging for patients with DS to receive timely care. These disruptions highlight the urgent need for adaptable and accessible dental care strategies that can withstand public health crises and ensure continuity of care for vulnerable populations.

Despite recognizing these challenges, there remains a significant gap in the literature regarding effective, evidence-based strategies to overcome these barriers. While some studies have explored some aspects of these barriers or offered insights into potential solutions [9], a comprehensive synthesis of the existing evidence is lacking. Such a synthesis is essential to inform future research, policy-making, and clinical practice, ultimately aiming to improve access to and the quality of dental care for patients with DS.

The increasing recognition of oral health as an integral part of overall health and well-being further underscores the importance of this research. Poor oral health is linked to serious systemic conditions [14], including cardiovascular disease, diabetes, and respiratory illnesses, particularly in vulnerable populations. For individuals with DS, these risks can be compounded by underlying physical and mental conditions such as an altered immune system or learning disabilities that can adversely affect oral health and the delivery of oral care [5].

Given the complexity and variability of barriers across different patient populations and healthcare systems, a scoping review is well-suited for mapping existing evidence and identifying research gaps. This approach enables a comprehensive understanding of the full spectrum of barriers to dental care for individuals with DS and an evaluation of the effectiveness of current strategies aimed at addressing these challenges.

The objective of this scoping review was to systematically examine, update, and synthesize the existing literature on the barriers to dental care experienced by patients with DS and to identify the strategies implemented to overcome these challenges. This review serves as a foundational step toward improving access to dental care for this vulnerable population by highlighting knowledge gaps and proposing directions for future research.

## 2. Methods

### 2.1. Study Design

This study was a scoping review, which is designed to map existing literature, provide a comprehensive overview of a given topic, and identify gaps in knowledge [15]. Scoping reviews are particularly useful for exploring complex and multifaceted subjects such as barriers to dental care for individuals with DS and the strategies to address these challenges. This review adhered to the Preferred Reporting Items for Systematic Reviews and Meta-Analyses (PRISMA) extension for Scoping Reviews [16] guidelines to ensure a rigorous and transparent methodological approach.

### 2.2. Research Question

The central research question guiding this scoping review was as follows: “What are the barriers to dental care for patients with DS, and what strategies have been implemented to overcome these barriers?” This question aimed to explore the obstacles faced by patients with DS in accessing dental care and to identify the solutions that have been proposed or implemented in various healthcare settings.

### 2.3. Eligibility Criteria

To ensure a comprehensive understanding of the barriers and strategies related to dental care for patients with DS, the review applied specific inclusion and exclusion criteria (Table 1). Eligibility criteria included qualitative, quantitative, case-controlled, cross-sectional, clinical, descriptive, randomized controlled trials, and mixed-methods studies that focused on barriers to dental care and strategies to overcome them in DS populations. We included studies that identified any type of barrier, such as financial, physical, systemic, or social barriers, as well as studies that described or evaluated strategies to overcome these barriers, including clinical interventions, policy changes, and training programs. Only peer-reviewed, English-language studies published within the last 10 years were considered. We limited the search to more recent publications since these should more accurately capture current dental issues for patients with DS.

Exclusion criteria included studies that did not specifically address dental care or did not focus on barriers and strategies relevant to patients with DS. Additionally, the review excluded studies that addressed barriers to dental care in the general population but did not focus on patients with DS. Review articles, editorials, case studies, research without human subjects, and opinion pieces were also excluded.

### 2.4. Information Sources

The literature search was conducted across several electronic databases, including PubMed, Scopus, and Web of Science. These databases were selected for their comprehensive coverage of medical, dental, and healthcare research, offering valuable insights into the barriers and strategies related to dental care for patients with DS.

### 2.5. Search Strategy

The study employed a systematic and comprehensive search strategy to identify relevant studies, utilizing keywords, MESH terms, and Boolean operators tailored to each database. Keywords included terms related to dental care, patients with DS, and strategies (Table 2).

### 2.6. Article Screening

The article screening process was conducted in two stages. First, two independent reviewers (A.G. and C.S.) screened the titles and abstracts for eligibility. Studies that met the inclusion criteria based on the title and abstract were then subjected to a full-text review to confirm their relevance. Any disagreements between reviewers during either stage were resolved through discussion and if the discordance could not be resolved, a third reviewer (M.H.) made the final decision. The selection process used EndNote version 21, a citation management software, to ensure efficiency and organization throughout the review.

### 2.7. Data Extraction

Data extraction was performed using a standardized form developed specifically for this review. The form was revised as needed to ensure it captured all relevant data accurately. Key data extracted from each study included study characteristics (e.g., author, year of publication, country of study, study design), population characteristics (e.g., age, sample size), identified barriers (e.g., financial, physical, systemic), strategies employed to overcome these barriers (e.g., clinical interventions, policy changes), and any reported outcomes related to the effectiveness of these strategies. To ensure accuracy and consistency, data extraction was conducted by two independent reviewers (A.G. and J.L.), with any discrepancies resolved through discussion or by consultation with a third reviewer (M.H.).

### 2.8. Data Analysis and Synthesis

The extracted data were analyzed and synthesized to identify and categorize barriers and strategies across different studies. A reference list of barriers to care was developed based on existing literature on barriers faced by children with disabilities. Using this framework, the authors systematically grouped similar barriers and strategies into key themes, focusing on identifying common challenges and effective solutions. Any discrepancies in categorization were resolved through discussion among all the authors to ensure consistency and accuracy.

### 2.9. Quality and Bias Assessment

While scoping reviews do not typically assess study quality in the same manner as systematic reviews, we implemented rigorous quality checks to evaluate the risk of bias, thereby enhancing the reliability and validity of our findings.

The risk of bias assessment was conducted using the Joanna Briggs Institute (JBI) Critical Appraisal Checklist for Analytical Cross-Sectional Studies, evaluating eight key domains: inclusion criteria, study population, exposure and outcome measurement, confounding control, statistical analysis, and result reporting [17]. Each study was rated as low, moderate, or high risk of bias based on how well these criteria were met. This systematic approach ensured a transparent and objective evaluation of study reliability.

## 3. Results

### 3.1. Study Selection

**Figure 1:**
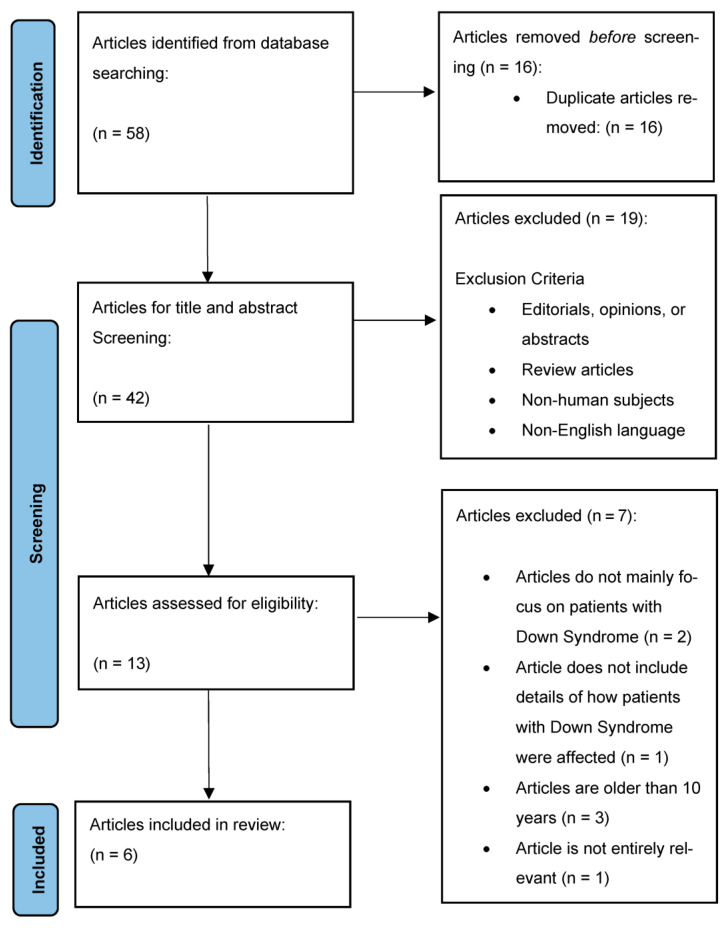
Flow diagram of article selection.

The initial search yielded a total of 58 articles. After removing duplicates, 42 articles remained for title and abstract screening. Following this initial screening, 13 studies were considered potentially relevant and underwent full-text review. Of these, six studies met the inclusion criteria for the review. The PRISMA flow diagram in Figure 1 outlines the study selection process.

### 3.2. Study Characteristics

The final sample consisted of six studies, published between 2015 and 2023. Two studies were conducted in the US and one each in Pakistan, Kuwait, Malaysia, and Saudi Arabia, reflecting a diverse geographical representation. All were cross-sectional studies and surveyed caregivers. The sample sizes ranged from 75 to 602 participants.

The review identified several themes regarding the barriers to dental care for children with DS and strategies to overcome these barriers (Table 3). These themes included difficulties maintaining oral hygiene, limited access to dental services, behavioral and sensory challenges, and recommended strategies for improving dental care.

**Table 3:** Key findings from the review of the included articles.

Author (Year)	Study Design	Study Location	Study Population	Sample Size	Barriers to Dental Care	Strategies to Overcome Barriers
Stein Duker et al. [18] (2020)	Cross-Sectional Study	Los Angeles, California	Caregivers of children 5–14 years old	372	Difficulty in brushing teeth regularly and effectively at home, difficulty finding a dentist willing and equipped to treat children with DS, high use of physical restraint or sedation during dental visits.	Increasing training for dental professionals in caring for special needs patients, improving access to dental care for children with DS, potentially specialized clinics.
Jawed et al. [19] (2021)	Cross-Sectional Study	Karachi, Pakistan	Caregivers of children 6–18 years old	196	Difficulty maintaining oral hygiene due to physical limitations, higher prevalence of dental caries among disabled children, and lack of access to proper dental care and treatment.	Implies the need for better oral hygiene practices and more accessible dental care for children with DS.
Shyama et al. [20] (2015)	Cross-Sectional Study	Kuwait	Caregivers of children with an average age of 10.8 for disabilities and 13.1 for DS	211 with disabilities and 97 with DS	Difficult to access an appointment, for children with DS, difficulty in cooperation, particularly highlighted by parents of disabled children. Fear, anxiety, and inability to cooperate with treatment.	Encouraging regular dental check-ups and preventive oral health care as part of a comprehensive national school oral health program.
Stein Duker et al. [20] (2022)	Cross-Sectional Survey	United States	Caregivers of children 5–14 years	367	Sensory over-responsivity, challenging behaviors, sensory sensitivities, and the need for full assistance to brush teeth.	Pediatric dentists should consider the role of sensory sensitivities and use sensory-based interventions when appropriate.
Wan Roselan et al. [21] (2023)	Cross-Sectional Study	Malaysia	Caregivers of children 0–16 years old	75	Lack of knowledge of dental needs, lack of access, lack of cooperation from the children.	Suggests the need for tailor-made comprehensive oral health care for children with DS.
Zahran et al. [22] (2023)	Cross-Sectional Study	Jeddah, Saudi Arabia	Caregivers of children 3–18 years old	602	The most commonly reported barrier was fear of the dentist (61.6%) followed by child uncooperativeness (37.8%) and treatment costs (27.8%), and accessibility issues.	Dentists require more training and education to facilitate access to dental care, but specific strategies not discussed.

### 3.3. Barriers to Dental Care Findings

#### 3.3.1. Difficulty in Maintaining Oral Hygiene

Several studies highlighted the significant challenge children with DS face in maintaining oral hygiene. Stein Duker et al. [18] surveyed the caregivers of children aged 5 to 14 years who reported difficulties brushing their teeth regularly and effectively at home. Similarly, Jawed et al. [19] reported that children with DS struggled with maintaining oral hygiene due to physical limitations, which led to a higher prevalence of dental caries. Additionally, Stein Duker et al. [20] identified sensory sensitivities and the need for full assistance with tooth brushing as barriers for children with sensory over-responsivity.

#### 3.3.2. Limited Access to Dental Services

Access to dental services emerged as a significant barrier across multiple studies. Stein Duker et al. [18] found that parents struggled to find dentists willing and equipped to treat children with DS. Shyama et al. [23] noted that obtaining dental appointments was particularly difficult for children with DS. Zahran et al. [22] also found that accessibility issues were a common problem, with parents reporting difficulty finding suitable dental care. Wan Roselan et al. [21] identified a lack of knowledge about dental needs and access to care as significant barriers. Access issue spanned several geographic locales including the US, Malaysia, and the Middle East.

#### 3.3.3. Behavioral and Sensory Challenges During Dental Visits

The studies consistently noted behavioral and sensory challenges as significant barriers to dental care. Stein Duker et al. [20] emphasized that sensory over-responsivity and challenging behaviors, such as requiring full assistance during dental procedures, were prevalent among children with DS. Shyama et al. [23] also highlighted fear, anxiety, and an inability to cooperate with treatment as significant barriers, particularly reported by parents of disabled children. Similarly, Zahran et al. [22] found that fear of the dentist and child uncooperativeness were key obstacles in accessing dental care.

### 3.4. Strategies to Overcome Barriers to Dental Care Findings

#### 3.4.1. Training and Education for Dental Professionals

Enhancing training and education for dental professionals was a consistent recommendation across studies to address barriers to care. Stein Duker et al. [18] and Zahran et al. [22] emphasized the need for more comprehensive training for dentists to handle special needs patients effectively, including those with DS. The studies suggest that by enhancing the skills and knowledge of dental professionals, there could be a significant reduction in the reliance on physical restraint or sedation, as well as an increase in the willingness of dentists to treat these populations.

#### 3.4.2. Development of Tailored Dental Care Approaches

Developing tailored dental care approaches was another key strategy identified to address the unique needs of children with DS. Stein Duker et al. [20] suggested using sensory-based interventions to accommodate children with sensory sensitivities. Similarly, Wan Roselan et al. [21] called for a comprehensive oral healthcare approach designed for children with DS, emphasizing the need for tailor-made solutions. These personalized approaches emphasize the importance of customizing dental care to meet the specific needs of children with DS, which can lead to better patient experiences and outcomes.

#### 3.4.3. Integration of Preventive Dental Programs

Integrating preventive dental programs into broader health initiatives was another proposed strategy to improve dental care accessibility for children with DS. Shyama et al. [23] recommended incorporating regular dental check-ups and preventive oral health care into a comprehensive national school oral health program. Such programs could facilitate early detection and intervention, reducing the need for more invasive treatments and helping to establish a routine of regular dental care from an early age.

### 3.5. Quality and Bias Assessment Findings

The risk of bias was assessed using the JBI Critical Appraisal Checklist for Analytical Cross-Sectional Studies, covering eight key domains: inclusion criteria, study setting, exposure and outcome measurement, confounding factors, statistical analysis, and reporting clarity [17]. The findings are summarized in Table 4.

Among the six studies assessed, three (Stein Duker et al. [18], Stein Duker et al. [20], and Zahran et al. [22]) demonstrated a low risk of bias, having met all critical quality assessment criteria. The remaining three studies (Jawed et al. [19], Shyama et al. [23], and Wan Roselan et al. [21]) exhibited a moderate risk of bias, primarily due to issues in handling confounding variables. Both Jawed et al. [19] and Shyama et al. [23] did not clearly identify confounding factors and lacked strategies to mitigate their impact. Wan Roselan et al. [21] had an even higher concern as it neither identified nor addressed confounding variables, which could introduce potential bias in its findings.

All studies used appropriate statistical analysis and presented conclusions that were well-supported by the data, ensuring robustness in their findings. However, the lack of confounding control in some studies suggests that their results should be interpreted with caution, especially when drawing comparisons or making generalized inferences.

**Table 4:** Risk of bias assessment.

Author (Year)	Clear Inclusion Criteria	Study Subjects and Setting	Exposure Measurement	Outcome Measurement	Confounding Factors Identified	Strategies for Confounding	Statistical Analysis	Appropriate Results and Conclusions	Overall Risk of Bias
Stein Duker et al. [18] (2020)	✔	✔	✔	✔	✔	✔	✔	✔	Low
Jawed et al. [19] (2021)	✔	✔	✔	✔	?	✘	✔	✔	Moderate
Shyama et al. [20] (2015)	✔	✔	✔	✔	?	✘	✔	✔	Moderate
Stein Duker et al. [20] (2022)	✔	✔	✔	✔	✔	✔	✔	✔	Low
Wan Roselan et al. [21] (2023)	✔	✔	✔	✔	✘	✘	✔	✔	Moderate
Zahran et al. [22] (2023)	✔	✔	✔	✔	✔	✔	✔	✔	Low

Note: Yes (✔)—Criteria met; No (✘)—Criteria not met; Unclear (?)—Insufficient information reported.

## 4. Discussion

This scoping review explored the barriers to dental care for children with DS and proposed strategies to address these challenges. The findings highlight several key barriers, including difficulties in maintaining oral hygiene, limited access to dental services, and behavioral and sensory problems during dental visits. By identifying these barriers, the review fills a gap in the literature and illustrates the need for multifaceted approaches to improve dental care for this population.

### 4.1. Barriers to Dental Care

Maintaining oral hygiene is a critical challenge for children with disabilities, as identified by several studies in this review. Stein Duker et al. [18] and Jawed et al. [19] reported that children with DS and other disabilities often experience difficulties in brushing their teeth effectively due to physical limitations and sensory sensitivities. These findings are consistent with a study by Quinzl et al. [24], who found that children with developmental disabilities often struggle with fine motor skills, making tasks like tooth brushing particularly challenging. Additionally, sensory sensitivities, as highlighted by Stein Duker et al. [20], can complicate oral hygiene routines since children may react negatively to the textures and sensations involved in tooth brushing. This challenge is exacerbated by the prevalence of cognitive impairments associated with DS that may limit their ability to understand and perform proper oral hygiene practices.

The implications of poor oral hygiene are significant, as inadequate oral care leads to a higher prevalence of dental caries, gingivitis, and other oral health problems that affect overall health and quality of life. The review emphasizes the need for tailored interventions that address these specific challenges. For example, modified toothbrushes with easier grips, educational programs for caregivers on proper oral hygiene techniques, and strategies to desensitize children to the sensory aspects of oral care may improve oral health. These approaches not only support better oral hygiene practices but also empower caregivers to manage their children’s dental health more effectively. Strategies published by Head Start for children with disabilities are one example of a caregiver-focused education strategy that may improve oral health in children with DS [25].

Limited access to dental services emerged as another major barrier identified by this review. Studies by Stein Duker et al. [18], Shyama et al. [23], and Zahran et al. [22] found that parents often face difficulties finding dental professionals who are willing and equipped to treat children with DS. This lack of access is compounded by a general shortage of dentists trained to handle special needs patients, as well as geographic disparities that influence the availability of specialized care. In particular, rural and underserved areas often face a more pronounced shortage of trained professionals, longer travel distances to specialty clinics, and fewer financial resources to support care. This finding aligns with research by Ajwa et al. [26], who found that less than half of dentists felt confident in treating patients with special needs. Moreover, Wan Roselan et al. [21] identified a lack of knowledge about dental needs and inadequate access to care as significant barriers, particularly in underserved regions. The interplay of cultural and social factors further shapes these disparities, as perceptions of disability, health-seeking behaviors, and systemic healthcare priorities vary across different countries and communities. For instance, in some regions, stigma surrounding disability may prevent families from seeking dental care, while in others, healthcare infrastructure limitations may make specialized treatment nearly inaccessible. This is consistent with the findings of Alfaraj et al. [27], who reported that geographic and economic factors exacerbate the lack of access to specialized dental services. Addressing access issues requires not only enhancing training for dental professionals but also implementing policy changes that facilitate access to specialized dental care. Policies should consider regional differences, ensuring equitable distribution of trained professionals and the development of culturally sensitive outreach programs to support families in navigating barriers to care.

Behavioral and sensory challenges during dental visits can complicate care for children with DS. Findings from Stein Duker et al. [20], Shyama et al. [23], and Zahran et al. [22] highlighted that fear, anxiety, and uncooperative behavior are common, often leading to the use of physical restraints or sedation. These behavioral and sensory challenges are not only distressing for the child but also pose ethical and safety concerns. For example, Kim et al. [28] found a disproportionately high use of sedation among children with special needs, raising concerns about a greater risk for adverse events and the appropriateness of such interventions. Sensory-adapted environments described by Nair et al. [29], such as dimmed lights and calming sounds, reduce anxiety and may improve cooperation in children with sensory sensitivities. Additionally, behavioral interventions, such as desensitization and positive reinforcement, can help mitigate fear and improve the dental experience for children with DS. By integrating these strategies, dental practices might accommodate the unique needs of these patients and reduce the reliance and risks of sedation.

### 4.2. Strategies to Overcome Barriers to Dental Care

Improved training and education for dental professionals are essential for overcoming many barriers. Both Stein Duker et al. [18] and Zahran et al. [22] emphasized that comprehensive training in special needs dentistry could reduce the use of physical restraints and increase the willingness of dentists to treat children with disabilities. Lynch et al. [30] argued that dental curricula should include more extensive training on managing patients with special needs. Training that focuses on communication strategies, understanding the behavioral cues of children with disabilities, and using non-pharmacological methods for reducing anxiety could significantly improve the quality of care provided.

Developing tailored dental care approaches is also crucial for addressing the unique needs of children with disabilities. Stein Duker et al. [20] and Wan Roselan et al. [21] advocated for dental care plans specifically designed to accommodate the unique needs of children with disabilities, such as sensory-based interventions for those with sensory sensitivities. These findings are supported by other research, such as van den Driessen et al. [31], which emphasizes the importance of individualized care plans that consider the patient’s medical, behavioral, and sensory needs. Tailored care approaches are essential for improving patient experiences and outcomes and ensuring that dental care is accessible and effective for children with diverse needs.

Integrating preventive dental programs into broader health initiatives is another strategy that can significantly improve dental care for children with disabilities. Shyama and colleagues [23] advocated for incorporating regular dental check-ups and preventive care into national school health programs, which aligns with recommendations from public health bodies like the American Academy of Pediatric Dentistry [32]. Preventive programs can help establish a routine of regular dental care and early intervention and are crucial for children with disabilities who may be more susceptible to dental diseases due to challenges in maintaining oral hygiene. By promoting a holistic approach to health care that includes oral health as a key component, preventive programs can reduce the burden of dental diseases and improve the overall well-being of children with DS.

### 4.3. Trends over Time in Access to Dental Care

The included studies span nearly a decade (2015–2023), providing an opportunity to explore whether barriers and strategies in dental care for children with DS have evolved over time. While our review did not initially focus on temporal trends, a closer examination of the data reveals some insights into changes in accessibility, professional training, and systemic challenges in dental care.

One key observation is that earlier studies, such as Jawed et al. [18] and Shyama et al. [20], primarily focused on structural barriers, including lack of trained professionals, financial constraints, and limited availability of specialized services. These studies emphasized geographic disparities and parental difficulties in obtaining routine dental care for children with DS.

In contrast, more recent studies, such as Stein Duker et al. [17,19] and Zahran et al. [21], highlighted an increasing focus on behavioral and sensory-related barriers alongside the persistence of systemic issues. These studies discussed the role of sensory sensitivities in dental care, suggesting a growing recognition of the need for tailored dental approaches and interventions that accommodate sensory processing challenges. Additionally, recent research identified progress in training programs for dental professionals, albeit inconsistently implemented across different regions.

However, despite some advancements in awareness and training, access to care remains a significant issue in both early and recent studies. Financial and insurance-related barriers persist, particularly in studies examining healthcare systems with limited specialized resources for individuals with disabilities. This suggests that while there have been efforts to improve awareness and professional competency, tangible improvements in dental access and equity for children with DS remain limited.

### 4.4. Practical Implementation of Recommendations

The findings of this review suggest several possibilities for improving dental care for children with DS. Enhancing training and education for dental professionals is crucial to equip dentists with the skills to handle special needs patients. This includes integrating DS-specific education into dental curricula, expanding continuing education, and providing hands-on clinical exposure through special education partnerships. Training should also cover sensory-based interventions, behavioral management techniques, and effective communication strategies for engaging with both children and their caregivers.

Developing tailored dental care approaches can further support the needs of children with DS. Implementing sensory-friendly dental environments, behavior-guided techniques such as visual schedules and desensitization therapy, and fostering interdisciplinary collaboration among dentists, therapists, and behavioral specialists can enhance patient comfort and cooperation.

For preventive care, integrating oral health into early intervention programs, expanding school-based and community dental initiatives, and strengthening insurance coverage can improve accessibility and reduce financial barriers. Embedding these efforts into broader health initiatives, such as school health programs, can promote early intervention, routine care, and a more inclusive, proactive approach to dental care for children with DS.

### 4.5. Strength and Limitations

This review has several strengths, including its comprehensive scope, which combines findings from multiple studies to provide a holistic view of the barriers to dental care for children with disabilities. By synthesizing diverse perspectives, the review offers insights into the interconnected nature of barriers and the need for multifaceted strategies to address them. However, there are several limitations. The review relies on the available literature, which may not fully represent all geographic regions or cultural contexts, potentially limiting the generalizability of the findings. Additionally, the review does not quantitatively assess the effectiveness of the proposed strategies. However, it suggests the need for future research to evaluate interventions. Moreover, the included studies were cross-sectional studies, limiting the understanding of causality and the long-term impact of these barriers and interventions over time. Also, many individuals with DS live into their 60s and no study examined adults with DS.

### 4.6. Future Research Directions

Future research should focus on quantitatively evaluating the effectiveness of the proposed strategies identified by this review to determine which interventions are most effective in improving dental care for children with disabilities. Longitudinal studies could provide insights into the long-term outcomes of tailored dental care approaches and preventive programs. They could assess whether policy changes, educational reforms, or technological advancements (e.g., teledentistry and sedation alternatives) have measurably improved access to dental care for this population. A more structured comparative analysis of pre- and post-2020 trends could provide deeper insight into whether healthcare policies and training interventions have led to measurable improvements or if access challenges have remained stagnant. Research should also explore barriers to dental care in underrepresented regions and among diverse populations to ensure that the findings are applicable across different cultural and socioeconomic contexts. Understanding these aspects will help develop more inclusive and effective strategies for improving dental care for all children with disabilities. Additionally, studies should consider the perspectives of caregivers and dental professionals to understand the practical challenges and barriers to implementing the proposed strategies.

## 5. Conclusions

This scoping review identified challenges in maintaining oral hygiene, limited access to specialized services, and behavioral and sensory difficulties during dental visits as key barriers to providing adequate dental care for children with DS. To overcome these barriers, the review highlights the need for better training for dental professionals, tailored care strategies, and integrated preventive programs. Implementing these strategies can enhance dental care access and contribute to the overall health and well-being of children with DS.

## Figures and Tables

**Table 1:** Inclusion and exclusion criteria.

Criteria	Inclusion	Exclusion
Population	Studies focusing on patients with DS.	Studies that do not specifically address patients with DS.
Focus	Studies identifying barriers to dental care (financial, physical, systemic, social) and/or strategies to overcome barriers.	Studies that do not address dental care or barriers and strategies related to patients with DS.
Study Designs	Qualitative, quantitative, and mixed-methods studies.	Review articles, editorials, and opinion pieces.
Interventions/Strategies	Studies describing or evaluating clinical interventions, policy changes, training programs, or other strategies aimed at overcoming dental care barriers.	Studies that do not include specific interventions or strategies for overcoming barriers in patients with DS.
Language	English-language studies only.	Non-English language studies.
Relevance to DS	Studies that specifically focus on barriers and strategies relevant to DS populations in dental care contexts.	Studies addressing barriers to dental care in the general population without a focus on patients with DS.

**Table 2:** Search strategy.

Database	Search Terms	Filters	Results	Date
PubMed	((“Down Syndrome” [Mesh]) OR (“Down Syndrome”)) AND ((“Barriers”) OR (“Barrier”) OR (“Limitations”) OR (“Limitation”)) AND ((“Oral care”) OR (“Oral healthcare”) OR (“Dent*”)) NOT (“review” [pt])	Human and English	20	28 February 2024
Scopus	TITLE-ABS-KEY ((“Down Syndrome”) AND ((“Barriers”) OR (“Barrier”) OR (“Limitations”) OR (“Limitation”)) AND ((“Oral care”) OR (“Oral healthcare”) OR (“Dent*”))) AND (EXCLUDE (DOCTYPE, “re”)) AND (LIMIT-TO (LANGUAGE, “English”))	English and Exclude Reviews	31	28 February 2024
Web of Science	(TI = ((“Down Syndrome”) AND ((“Barriers”) OR (“Barrier”) OR (“Limitations”) OR (“Limitation”)) AND ((“Oral care”) OR (“Oral healthcare”) OR (“Dent*”)))) OR AB = ((“Down Syndrome”) AND ((“Barriers”) OR (“Barrier”) OR (“Limitations”) OR (“Limitation”)) AND ((“Oral care”) OR (“Oral healthcare”) OR (“Dent*”))	English and Exclude Reviews	7	28 February 2024
